# Reprogramming the diseased liver: antioxidant-engineered mRNA nanoparticles as microenvironment modulators in MAFLD

**DOI:** 10.1097/IN9.0000000000000083

**Published:** 2026-05-19

**Authors:** Matteo Ghiringhelli, Lior Zangi

**Affiliations:** 1 Cardiovascular Research Institute, Icahn School of Medicine at Mount Sinai, New York, NY, USA; 2 Department of Genetics and Genomic Sciences, Icahn School of Medicine at Mount Sinai, New York, NY, USA; 3 Black Family Stem Cell Institute, Icahn School of Medicine at Mount Sinai, New York, NY, USA

**Keywords:** MAFLD, mRNA therapeutics, lipid nanoparticles, TCPTP, STAT signaling, oxidative stress, hepatocellular carcinoma, immune microenvironment

## Abstract

Metabolic dysfunction–associated fatty liver disease (MAFLD) is a leading cause of hepatocellular carcinoma (HCC), driven in part by oxidative stress–induced immune dysfunction that limits the efficacy of immunotherapy. In a recent study, Yu et al developed a vitamin E–incorporated lipid nanoparticle designed to enhance hepatocyte‑specific mRNA delivery while simultaneously buffering oxidative stress in the hepatic microenvironment. By restoring the activity of the redox‑sensitive phosphatase T‑cell protein tyrosine phosphatase and suppressing signal transducer and activator of transcription (STAT) signaling, this strategy improves metabolic homeostasis, reduces inflammatory signaling, and enhances responsiveness to immune checkpoint blockade. These findings illustrate how rational nanoparticle engineering can integrate therapeutic delivery with microenvironmental reprogramming in chronic metabolic disease.

Metabolic dysfunction–associated fatty liver disease (MAFLD) has become a major global driver of hepatocellular carcinoma (HCC), paralleling the increasing prevalence of obesity and metabolic syndrome worldwide ^[1]^. Beyond simple steatosis, the progression to metabolic‑associated steatohepatitis (MASH) involves lipid accumulation, oxidative stress, and immune dysregulation that reshape the hepatic microenvironment into a pro‑tumorigenic niche ^[2]^. Importantly, this inflammatory metabolic landscape can also influence therapeutic outcomes. Evidence indicates that HCC arising in nonalcoholic steatohepatitis (NASH) and MASH livers responds less favorably to immune checkpoint blockade because of altered immune surveillance and dysfunctional cytotoxic T‑cell responses ^[3]^. Within this context, the study by Yu et al proposes an innovative strategy for mRNA therapeutics that goes beyond conventional delivery optimization ^[4]^. Instead of treating the nanoparticle as a neutral carrier, the authors engineered a lipid nanoparticle (LNP) capable of actively modifying the oxidative hepatic environment while delivering therapeutic mRNA. Their approach centers on the T‑cell protein tyrosine phosphatase (TCPTP)–signal transducer and activator of transcription (STAT) signaling axis, an important regulatory pathway linking metabolic stress, inflammatory signaling, and tumor progression. TCPTP normally acts as a negative regulator of STAT1 and STAT3 signaling, thereby restraining inflammatory transcriptional programs and maintaining metabolic homeostasis ^[4]^. However, the catalytic cysteine residue within protein tyrosine phosphatases is highly sensitive to oxidation. Reactive oxygen species can convert this cysteine into an inactive oxidized state, thereby sustaining downstream signaling pathways ^[5]^. In the MAFLD liver, excessive lipid accumulation promotes oxidative stress and lipid peroxidation, creating conditions that favor phosphatase inactivation and persistent STAT signaling ^[3,4]^. Yu et al show that MAFLD liver samples exhibit increased STAT1/3 phosphorylation together with reduced functional TCPTP activity, suggesting that disruption of this regulatory axis contributes to disease progression ^[4]^. These observations highlight a broader challenge for mRNA therapeutics in chronic metabolic disease. Many lipid nanoparticle systems have been optimized for vaccination or short‑term protein replacement, where tissue homeostasis is relatively stable ^[6]^. In contrast, MAFLD represents a chronically inflamed and oxidized environment that can compromise protein activity and exacerbate inflammatory signaling. Consequently, delivery strategies that ignore the biochemical context of diseased tissues may fail to achieve their full therapeutic potential. To address this limitation, Yu et al redesigned the helper lipid component of the nanoparticle by incorporating a vitamin E–derived phosphatidylcholine, generating a new formulation termed Def‑LNP ^[4]^. Vitamin E is a lipid‑soluble antioxidant that has long been investigated as a therapeutic strategy in non‑alcoholic steatohepatitis because of its ability to reduce oxidative stress and lipid peroxidation ^[7]^. By embedding this antioxidant functionality directly into the nanoparticle architecture, the authors created a delivery platform that can simultaneously transport therapeutic RNA and mitigate oxidative stress within hepatocytes. From a chemical perspective, this design is particularly interesting because vitamin E acts as a chain‑breaking antioxidant in biological membranes. Tocopherols donate a hydrogen atom to lipid peroxyl radicals, thereby terminating radical propagation reactions that occur during lipid peroxidation ^[8]^. In hepatocytes overloaded with lipids, such radical chain reactions can generate reactive aldehydes and reactive oxygen species capable of modifying redox‑sensitive cysteine residues in signaling proteins. Because TCPTP relies on a catalytic cysteine for phosphatase activity, it is especially susceptible to oxidative inactivation. The incorporation of vitamin‑E‑derived lipids into the nanoparticle may therefore locally buffer oxidative reactions during cellular uptake and endosomal escape, helping preserve TCPTP activity and maintain downstream regulatory signaling. Consistent with this mechanistic rationale, Def‑LNP nanoparticles demonstrated improved hepatocyte targeting, reduced uptake by inflammatory Kupffer cells, and prolonged hepatic mRNA expression compared with conventional LNP formulations ^[4]^. Most importantly, the antioxidant lipid environment reduced the formation of oxidized phosphatase species and enabled sustained TCPTP activity following mRNA delivery. The restored phosphatase function suppressed STAT1/3 signaling and decreased inflammatory cytokine production while improving markers of hepatic lipid metabolism. At the tissue level, these molecular effects translated into physiological benefits. Def-LNP-mediated TCPTP restoration alleviated steatohepatitis, reduced oncogenic signaling pathways, and improved responses to immunotherapy in experimental models ^[4]^. Transcriptomic analyses revealed broad remodeling of pathways associated with inflammation, lipid metabolism, and chemokine signaling, suggesting that correcting the TCPTP–STAT axis can reshape both metabolic and immunological aspects of the diseased liver environment. The broader implication of this work is conceptual. Nanoparticle delivery systems are often designed primarily to maximize transfection efficiency, yet their biochemical composition inevitably interacts with cellular metabolism and immune signaling. By incorporating antioxidant functionality directly into the nanoparticle structure, the Def‑LNP platform illustrates how delivery systems can be engineered to counteract disease‑specific biochemical stressors rather than simply tolerate them. An additional translational consideration is the potential hepatic burden associated with repeated LNP administration in chronic liver disease. Because many systemically administered LNP formulations preferentially accumulate in the liver, repeated dosing may exacerbate hepatocellular stress, innate immune activation, or inflammatory signaling, particularly in tissues already affected by steatosis, oxidative injury, and metabolic inflammation. This issue is especially relevant for MAFLD and MASH, where therapeutic strategies may require sustained or repeated intervention rather than a single short-term exposure. In this regard, the Def-LNP design described by Yu et al may offer an important advantage (Figure 1). By incorporating vitamin E–derived antioxidant functionality into the nanoparticle itself, the platform is not only optimized for mRNA delivery but also engineered to buffer disease-associated oxidative stress. This dual activity may help reduce the inflammatory and oxidative liabilities often associated with repeated lipid nanoparticle exposure, thereby improving the feasibility of RNA-based interventions in chronically diseased hepatic tissue. Because MAFLD‑associated HCC continues to rise globally, therapeutic strategies that address both metabolic dysfunction and immune dysregulation will become increasingly important. The work by Yu et al highlights a promising direction in which rational nanoparticle engineering enables RNA therapeutics not only to deliver genetic information but also to reshape the pathological microenvironment in which that information must function.

**Figure 1.**
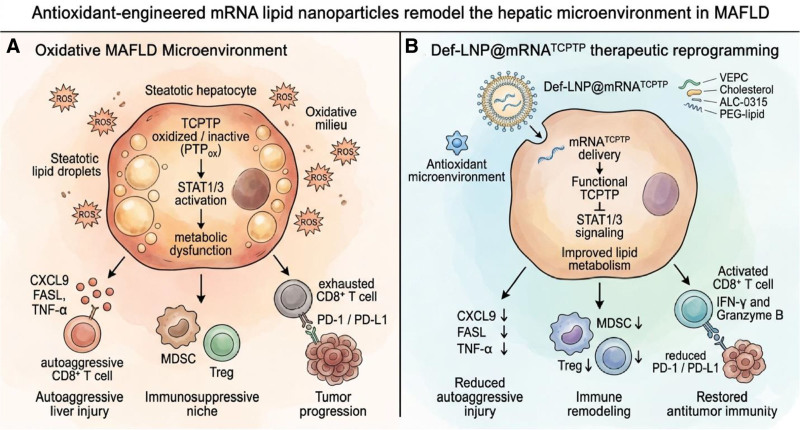
**Antioxidant-engineered mRNA lipid nanoparticles remodel the hepatic microenvironment in metabolic dysfunction–associated fatty liver disease (MAFLD).** (A) Oxidative MAFLD microenvironment. MAFLD is characterized by lipid accumulation in hepatocytes and a highly oxidative hepatic milieu. Reactive oxygen species (ROS) promote oxidative inactivation of the redox-sensitive phosphatase T-cell protein tyrosine phosphatase (TCPTP), resulting in sustained activation of STAT1/3 signaling and metabolic dysfunction. This pathological signaling state contributes to inflammatory chemotaxis and immune dysregulation within the liver. Elevated levels of chemokines and inflammatory mediators (including CXCL9, FASL, and TNF-α) promote auto-aggressive CD8^+^ T-cell–mediated liver injury, while recruitment of immunosuppressive populations such as myeloid-derived suppressor cells (MDSCs) and regulatory T cells (Tregs) contributes to an immunosuppressive niche. In parallel, antitumor CD8^+^ T cells exhibit an exhausted phenotype characterized by PD-1/PD-L1 signaling, facilitating immune evasion and hepatocellular carcinoma progression. (B) Def-LNP@mRNA TCPTP therapeutic reprogramming. Antioxidant-engineered lipid nanoparticles (Def-LNPs) incorporating a vitamin E–derived phospholipid (VEPC) deliver mRNA TCPTP to hepatocytes while simultaneously buffering oxidative stress within the hepatic microenvironment. Restored expression of functional TCPTP suppresses STAT1/3 signaling, improving hepatocyte metabolic homeostasis and lipid metabolism. This metabolic reprogramming attenuates inflammatory chemotaxis and reduces expression of CXCL9, FASL, and TNF-α, thereby limiting autoaggressive CD8^+^ T-cell–mediated liver injury. Concurrently, the suppressive immune niche is diminished through reduced MDSC and Treg abundance, while antitumor CD8^+^ T cells regain effector function with increased IFN-γ and granzyme B activity and reduced PD-1/PD-L1 signaling. Collectively, Def-LNP–mediated delivery of mRNA TCPTP restores hepatic immune surveillance and suppresses tumor progression by remodeling both the metabolic and immunological landscape of the MAFLD liver. Graphic created with Gemini Nano Banana 2 and edited in Adobe Illustrator by the authors. CXCL9, chemokine; FASL, Fas ligand; IFN-γ, interferon-gamma; PD-1/PD-L1, programmed cell death protein 1/programmed death–ligand 1; TNF-α, tumor necrosis factor-alpha.

## Conflicts of interest

The authors declare that they have no conflicts of interest.

## Funding

L.Z. is supported by an NIH grant (R01HL142768).
